# Altered Composition of Bone as Triggered by Irradiation Facilitates the Rapid Erosion of the Matrix by Both Cellular and Physicochemical Processes

**DOI:** 10.1371/journal.pone.0064952

**Published:** 2013-05-31

**Authors:** Danielle E. Green, Benjamin J. Adler, Meilin Ete Chan, James J. Lennon, Alvin S. Acerbo, Lisa M. Miller, Clinton T. Rubin

**Affiliations:** 1 Department of Biomedical Engineering, Stony Brook University, Stony Brook, New York, United States of America; 2 Photon Sciences Directorate, Brookhaven National Laboratory, Upton, New York, United States of America; University of Notre Dame, United States of America

## Abstract

Radiation rapidly undermines trabecular architecture, a destructive process which proceeds despite a devastated cell population. In addition to the ‘biologically orchestrated’ resorption of the matrix by osteoclasts, physicochemical processes enabled by a damaged matrix may contribute to the rapid erosion of bone quality. 8w male C57BL/6 mice exposed to 5 Gy of Cs^137^ γ-irradiation were compared to age-matched control at 2d, 10d, or 8w following exposure. By 10d, irradiation had led to significant loss of trabecular bone volume fraction. Assessed by reflection-based Fourier transform infrared imaging (FTIRI), chemical composition of the irradiated matrix indicated that mineralization had diminished at 2d by −4.3±4.8%, and at 10d by −5.8±3.2%. These data suggest that irradiation facilitates the dissolution of the matrix through a change in the material itself, a conclusion supported by a 13.7±4.5% increase in the elastic modulus as measured by nanoindentation. The decline in viable cells within the marrow of irradiated mice at 2d implies that the immediate collapse of bone quality and inherent increased risk of fracture is not solely a result of an overly-active biologic process, but one fostered by alterations in the material matrix that predisposes the material to erosion.

## Introduction

Radiation exposure has become a large health concern due to factors including the recent reactor failures at Fukushima Daiichi, the high clinical doses patients receive for radiotherapy, and the exposure astronauts receive during extended space missions [1], [2], [3]. In addition to radiation’s destruction of the bone marrow and the resident hematopoietic and mesenchymal stem cell populations, this exposure – whether intentional or otherwise - leads to the devastation of bone architecture, thereby increasing a person’s lifetime risk of fracture [4], [5], [6]. While the mechanism of bone loss following exposure is presumed to be a biological process mediated by elevated osteoclast activity [7], [8], considering the extensive destruction of the precursor population, it is possible that – to some degree – the bone loss is achieved independent of “biology” via an acellular process, perhaps via the physicochemical dissolution of a damaged bone matrix.

The bone matrix is composed of organic components, including collagen type-I and non-collagenous proteins, and an inorganic component comprised of carbonated hydroxyapatite. Damage to either the inorganic or organic constituents of the matrix drastically compromise bone quality, as evidenced by the severe decline in the bone’s mechanical properties following irradiation [9], [10]. This dose-dependent decline in mechanical properties includes reductions in bone strength, ductility, and fracture resistance, with higher exposure to radiation directly correlating to poorer bone quality [11], [12].

As demonstrated *ex vivo,* irradiation also compromises bone strength. In bone allograft transplantation, a method commonly employed in orthopedic bone reconstruction, the bone graft will typically be irradiated at dose exposures greater than 25 kGy to minimize the potential for transmittance of diseases from donor to recipient [13]. Even in acute, *ex vivo* exposures, these high doses directly reduce bone’s material properties [14], belying not only a compromised material central to surgery, but suggesting a very real risk that radiation poses to the skeleton during both intentional or unintentional exposures. And while *ex vivo* reductions in material properties occur independent of biologic processes, it is also obvious, but important to point out that this reduction occurs independent of reductions in bone morphology (e.g., bone volume fraction, trabecular number, etc.).

While cancer patients typically receive somewhat lower doses of radiation prior to bone marrow transplantation (∼12 Gy) [15], this exposure can reach as high as ∼66 Gy in localized regions targeted to ablate tumors [16], predisposing these specific regions to accelerated bone loss and elevated risk of fracture [4], [5], [6]. Despite the marked depletion of the bone marrow progenitor population within even two days of irradiation exposure, which include the hematopoietic precursors to osteoclasts [17], bone loss in these clinical cases are typically presumed to result from elevated osteoclast activity. However, if the hematopoietic population is crippled following irradiation, it is difficult to attribute the almost instantaneous decline in bone architecture - realized within 10 days [17]- solely to bio-mediated bone resorption. We propose, therefore, that removal of the matrix following irradiation is facilitated by damage to the bone matrix itself. Testing of this hypothesis has been enabled by recent advances in quantitative microscopy which allow a full characterization of the organic and inorganic constituents of bone, as well as new advances in material property characterization, which allow a full assessment of the mechanics of the matrix.

**Figure 1:**
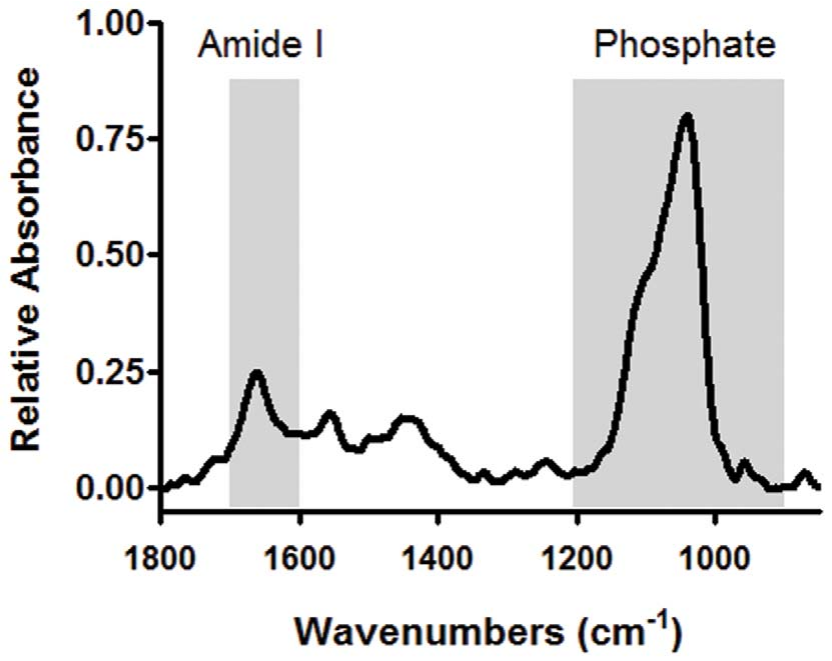
An infrared spectrum of a typical trabecular bone sample collected using Fourier transform infrared microscopy. The highlighted peaks, falling between 900–1200 cm^−1^ and 1600–1700 cm^−1^, correspond to the phosphate and amide-I peaks, respectively, which are used to calculate the level of tissue mineralization.

Fourier transform infrared imaging (FTIRI) can be used to map the chemical composition of bone [18], [19], [20]. Defined as the phosphate/protein ratio, FTIRI can assess the level of tissue mineralization by integrating the protein amide I peak falling between 1600 cm^−1^–1700 cm^−1^ and the phosphate peak (ν_1_,ν_3_ PO_4_^3–^) falling between 900 cm^−1^–1200 cm^−1^ (Fig. 1). Any alteration in the content of protein or phosphate will thereby affect the total level of tissue mineralization, a critical correlate to the overall mechanical properties of bone [19], [21]. In addition to characterizing key components of tissue mineralization, reflection based FTIRI is capable of identifying independent changes to the bone matrix due to alterations in the organic and inorganic components of the matrix. For instance, a shift in the phosphate intensity infers an alteration to the stoichiometric properties of hydroxyapatite crystals within the mineralized matrix, while a change in amide I intensity implicates a change in the chemical composition of the protein structure, primarily collagen type-I. FTIRI can also be used to assess crystallinity, a measure of the size and shape of crystallites. The spatial resolution of FTIRI mapping facilitates spatial mapping of the chemical composition in different regions of the bone matrix, allowing for the identification of sites most susceptible to radiation-induced destruction.

While FTIRI is a method used to analyze chemical composition, nanoindentation can be used to characterize the surface mechanical properties of materials on the nano-micron scale [22], [23]. Typically a diamond probe penetrates into the surface of the tissue, and the material’s elastic properties such as hardness and elastic modulus can be determined from the applied load and depth of sample penetration [23]. A benefit of nanoindentation is its site specificity for indent placement [24], and therefore the mechanical properties of bone in one region can easily be compared to the same region of another sample. Other methods such as whole bone testing often return the bone’s bulk material properties which do not necessarily relate to trabecular bone quality, thereby making nanoindentation the most ideal method to determine trabecular bone’s elastic properties.

We hypothesize that the rapid bone loss caused by irradiation is a complex product of biological resorption driven by increased osteoclast activity, and physicochemical-based erosion enabled by damage to the matrix that advances independent of the biology of the system. In this study, we investigate the capacity of sub-lethal doses of radiation to alter the chemical and physical properties of the bone matrix in young adult C57BL/6 mice. We attempt to distinguish between the biological and physicochemical processes, although not mutually exclusive, which contribute to bone loss, showing that these processes play not only additive, but independent roles in the destruction of bone quantity and quality.

## Materials and Methods

### Ethics Statement

All animal procedures were reviewed and approved by Stony Brook University’s Institutional Animal Care and Use Committee, ID # 0067.

### Irradiating Mice and Tissue Harvest

Six week old male C57BL/6 mice (Jackson Laboratories) were acclimated for 2w prior to the start of the study and fed food and water *ad libitum*. At 8w of age, mice were placed in an irradiation chamber and subjected to 0.6 Gy/min Cs^137^ γ-irradiation for 8.4 min, reaching a 5 Gy cumulative dose. Age-matched control mice were placed into the inactive irradiator for the identical period of time. At 2d, 10d or 8wk following radiation exposure, one third of mice in the irradiated and control groups were anesthetized with isoflurane and sacrificed using cervical dislocation (n = 7–8 per group). The left tibiae were extracted and placed into 70% ethanol and stored at −20°C for preservation, and blood was removed from the mice via cardiac puncture at the time of sacrifice. The right tibiae were extracted and bone marrow was flushed with Dulbecco’s Modified Essential Medium containing 2% fetal bovine serum (Invitrogen), 10 mM HEPES Buffer (Gibco), and 1% Penicillin & Streptomycin (Gibco). The right humerus was extracted, placed into phosphate buffered saline, and stored at −20°C for preservation. Total cell numbers were quantified using a Scepter (Millipore) cell counter following erythrocyte lysis.

### Trabecular and Cortical Bone Architecture Using Micro-Computed Tomography (microCT)

In order to analyze the microarchitecture of trabecular and cortical bone following irradiation, the mice tibiae were scanned *ex vivo* using a microCT scanner (µCT 40; Scanco Medical) with a 12 µm isotropic voxel size. The x-ray source voltage was 55 kVp, source current was 145 µA, and integration time was 300 ms. Using well defined automated analysis scripts [25], an 840 µm region of trabecular bone was analyzed approximately 300 µm distal to the growth plate in order to measure trabecular tissue mineral density (Tb.TMD). A 600 µm region of cortical bone was assessed in the midshaft to measure the cortical thickness (Ct.Th), cortical area (Ct.Ar), total area (Tt.Ar), and cortical area fraction (Ct.Ar/Tt.Ar) of the tibiae.

### Bone Sample Embedding

Before embedding the undecalcified tibiae in poly(methyl methacrylate) (PMMA), the bones were serially dehydrated in 70%, 95%, and 100% isopropanol and cleared in petroleum ether. Samples were then infiltrated with PMMA solution containing 15% n-butyl phthalate (Sigma), 85% methyl methacrylate (Fisher), and 2% w/v benzoyl peroxide (Aldrich). The tibiae were then placed into 20 ml polyethylene scintillation vials containing hardened PMMA, covered with fresh PMMA solution, and stored in a 37°C water bath until the PMMA solidified.

### Fourier Transform Infrared Imaging

Fourier transform infrared imaging (FTIRI) provides a high resolution spatial assessment of the mineral composition and collagen cross-linking in the trabecular bone [26]. Cross-sections of bone from irradiated and control mice were first ground with abrasive silicon carbide papers using finer grits with decreasing particle sizes of 600, 800, and 1,200, then polished with a diamond suspension of 3 µm followed by 1 µm, 0.25 µm, and 0.05 µm, and finally sonicated in water to remove any remaining debris. FTIRI data collection were performed on the PMMA embedded and polished tibiae sample blocks using a Hyperion 3000 FTIR microscope (Bruker Optics) equipped with a 64×64 element focal plane array detector at beamline U10B of the National Synchrotron Light Source at Brookhaven National Laboratory. FTIRI data were collected in a reflection geometry over a range of 850–3900 cm^−1^ with a spectral resolution of 8 cm^−1^ and a pixel resolution of 5 µm. A total of 128 scans were collected per pixel, with a reflective gold slide used as a background calibration prior to scanning samples. The resultant reflection spectra were processed and transformed into absorbance spectra using Kramers-Kronig transformation [27].

Using Cytospec 1.4.03 (CytoSpec Inc.), the absorbance spectra of the trabecular bone in the metaphysis first underwent a linear baseline correction from 900 cm^−1^ to 1800 cm^−1^. The protein of amide I (1600 cm^−1^–1700 cm^−1^), amide II (1510 cm^−1^–1595 cm^−1^), crystallinity (1033 cm^−1^–1037 cm^−1^)/(1023 cm^−1^–1027 cm^−1^), and phosphate (900–1200 cm^−1^) peaks were integrated and the mineral/protein ratio was calculated [19], [28], [29]. Amide I and amide II are both characteristic spectral regions for collagen type-I. To avoid any confounding contribution of a PMMA artifact into the bone spectra, as well as pixels on the bone surface with only partial bone contribution, a PMMA mask was set in place, and pixels with the characteristic PMMA absorbance peak between 1710 cm^−1^ and 1775 cm^−1^ were removed from the analysis. This mask removes 10 µm−15 µm of data from the bone surface.

To determine the difference in phosphate, protein, and mineralization at the periphery of each trabecular element compared to the average content throughout the strut, all FTIRI data were removed from each independent trabeculae with the exception of the outermost pixel thereby leaving only an outline of the trabeculae, which represents phosphate, protein, or mineralization of the bone surface.

### Nanoindentation

Following FTIRI, load controlled nanoindentation (Hysitron Triboindenter) was performed using a Berkovich tip in the same trabecular region analyzed for the bone’s chemical composition. The tip shape function was calibrated using fused silica, which is elastically isotropic. A three segment load function was used for indenting, starting with a constant loading rate of 67 µN/s for 15 s, followed by a 10s hold period, and completed by a 67 µN/s unloading rate for 15s, thereby having a 1000 µN peak force, and 20 indentations were made per sample. These 20 points were selected using an optical microscope with a precision stage (500 nm accuracy). The elastic properties were calculated between 50% and 95% of the initial unloading curve and the Oliver-Pharr method was used to calculate elastic modulus and hardness [22], [30]. Elastic modulus (E) and hardness (H) of the bone were defined as:
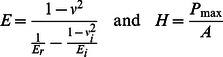
where *v_i_* and *v* are the Poisson’s ratio of the indenter and trabecular bone assumed to be 0.07 and 0.25, respectively. E_i_ is the reduced elastic modulus of the indenter assumed to be 1140 GPa and E_r_ is the reduced elastic modulus calculated as 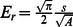 where S is the contact stiffness, A is the contact area at maximum load, and P_max_ is the maximum indentation load.

### Mechanical Loading

The right humeri of 10d mice were thawed to room temperature, fixed horizontally within the supports of the MTS, and three point bending strength was assessed. The supports spanned 6 mm. A 0.1 N of static preload was applied to the humeri, and then they were loaded to failure at 0.10 mm/s. The ultimate force and stiffness were evaluated from the force-displacement curves.

### Bone Marrow Histology

Bones embedded in PMMA were sectioned using a microtome (Leica) at 6 µm, the PMMA sections were deplasticized in acetone for 1 hr, and stained with Modified Wright Giemsa (Sigma) for 1 min, rinsed with water, and then imaged under a light microscope (Zeiss). The percentage of the bone marrow occupied by adipocytes in the proximal tibiae below the growth plate was quantified using ImageJ software (NIH).

### Cell Proliferative Ability Using Colony Forming Cell Assay

The harvested bone marrow from the right tibiae of control and irradiated mice were cultured at 1×10^4^ cells per 35 cm plate in methylcellulose based media, and at 14d following the sacrifice, the total number of colonies formed was quantified according to the manufacturer’s protocol (R&D Systems). The total number of colonies was identified based on morphology as either colony forming unit-granulocyte macrophage (CFU-GM) or colony forming unit-granulocyte, erythrocyte, macrophage, megakaryocyte (CFU-GEMM).

### Osteoclast Activity

The level of osteoclast activity was assayed by the serum concentration of tartrate-resistant acid phosphatase (TRAP5b) assay (MouseTRAP; Immunodiagnostic Systems) according to the manufacturer’s protocols.

### Collagen I Breakdown

The breakdown of the organic matrix was quantified using plasma samples diluted in PBS. The type-I Collagen was quantified using the Mouse Cross Linked C-telopeptide of Type-I Collagen (CTX-I) Elisa kit according to the manufacturer’s protocol (MyBioSource.com). The average of the optical density values was fit to the standard curve and multiplied by the dilution factor to determine the average concentration of CTX-I in the plasma.

### Statistics

All data are presented as mean ± standard deviation. Differences between control and irradiated mice are determined using an unpaired sample t-test, where p<0.05 is considered statistically significant. To determine the difference between the mineralization in the center of a trabecular strut as compared to its surface, a paired sample t-test is used, where p<0.05 is considered significant.

## Results

### Rapid Destruction of Trabecular Bone Architecture Following Radiation Exposure but Cortical Bone Remained Uncompromised

As we previously reported, radiation exposure led to significant loss of trabecular bone as early as 10d following irradiation, as shown by the −41±12% and −33±4% decline in bone volume fraction and trabecular number, and 52±8% increase in trabecular separation compared to the control [17]. By 8w, bone volume fraction and trabecular number showed no evidence of improved trabecular morphology, despite the trend of a 2.7±3.5% increase in Tb.TMD (p = 0.09; Table 1). In contrast, no bone loss was evident in cortical bone at any point measured throughout the study as seen by no differences in Tt.Ar, Ct.Ar, Ct.Th, and Ct.Ar/Tt.Ar between the irradiated and control mice (Table 1).

**Table 1:** Trabecular and cortical bone parameters in the tibiae of irradiated mice compared to the age-matched control at 2d, 10d, and 8w.

		Tb.TMD (mg/cc)	Ct.Th (mm)	Tt.Ar (mm^2^)	Ct.Ar (mm^2^)	Ct.Ar/Tt.Ar
**2 Day**	Control (n = 7)	769±16	0.19±0.02	0.57±0.07	0.57±0.07	0.994±0.001
	Irradiated (n = 8)	766±15	0.18±0.01	0.54±0.07	0.53±0.07	0.993±0.002
	% Difference	−0.3±2.0	−6.6±7.0	−5.6±12.5	−5.7±12.5	−0.12±0.22
	P-value	0.79	0.22	0.40	0.55	0.19
**10 Day**	Control (n = 8)	766±14	0.19±0.01	0.56±0.04	0.55±0.04	0.992±0.004
	Irradiated (n = 8)	773±11	0.19±0.01	0.57±0.06	0.57±0.06	0.994±0.000
	% Difference	1.0±1.4	3.3±7.0	2.5±10.4	2.7±10.4	0.13±0.04
	P-value	0.24	0.33	0.58	0.56	0.40
**8 Week**	Control (n = 8)	801±8.9	0.20±0.01	0.59±0.04	0.58±0.04	0.994±0.001
	Irradiated (n = 7)	823±28	0.21±0.01	0.60±0.02	0.60±0.02	0.994±0.001
	% Difference	2.7±3.5	3.2±4.6	2.4±3.5	2.4±3.5	−0.05±0.06
	P-value	0.09	0.15	0.38	0.39	0.13

By 10d following irradiation, although there was a deficit to the trabecular bone architecture compared to non-irradiated control, no significant differences were apparent in the cortical bone between the irradiated mice and the age-matched control.

### Decline in Trabecular Bone Mineralization Following Irradiation

**Figure 2:**
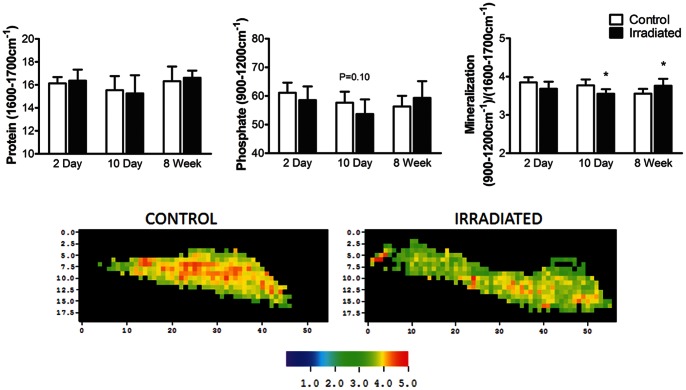
Trabecular bone chemical composition using FTIRI. A) Level of mineralization as a proportion of the inorganic to organic matrix, in the trabecular bone of irradiated mice compared to control at 2d, 10d, and 8w following irradiation. *p<0.05 compared to age-matched control. B) A typical FTIRI heat map of the level of mineralization in a control (left) and an irradiated (right) trabecular strut at 2d. Increased intensity corresponds to increased degree of mineralization.

Evident at 2d, there is a trend toward a −4.3±4.8% decline in mineralization of the trabecular struts in the irradiated mice as compared to control (p = 0.07), which further falls by 10d to −5.8±3.2% (p<0.01; Fig. 2). A decline in the mineral/matrix ratio appears to occur through a decrease in the phosphate content within the bone matrix (Fig. 2). Irradiated mice show a −4.2±7.8% (NS) and −6.8±8.8% (p = 0.10) decline in phosphate content at 2d and 10d compared to age-matched control, whereas no significant changes were evident in protein level between the irradiated mice and their control at either 2d or 10d, respectively. Even with the decline in mineralization, crystallinity, a measure of apatite size and shape, shows no differences between the irradiated mice and control at 2d (Control = 1.48±0.07, Irradiated = 1.58±0.16; p = 0.12) or 10d (Control = 1.53±0.11, Irradiated = 1.59±0.14; p = 0.40). By 8w, although the trabecular BV/TV in the tibiae remained −45±9% below age-matched control [17], the level of mineralization in the irradiated mice tibiae had fully recovered, and were 5.8±5.1% (p<0.05) greater than the control, a finding consistent with the increase in Tb.TMD seen using micro-CT. Crystallinity of the irradiated mice though was −12±7% lower than the age-matched control (p<0.05).

In contrast to those measures made in the trabecular compartment, at 10d post-irradiation, FTIRI showed no change in the chemical composition of the cortical bone, confirming the micro-CT analysis which indicated no loss of bone in this region (Table 1). At 10d, mineralization in cortical bone was 4.3±0.2 and 4.4±0.2 in the control and irradiated bones, respectively (p = 0.47). These data also indicated that bone in the trabecular compartment of the control was −11±3% less mineralized than the cortical bone of the control, with phosphate and protein levels −16±3% and −5±3% lower than cortical bone, respectively.

### Decline in Phosphate and Protein Following Irradiation was Uniform Across the Trabecular Strut Rather than Localized to the Surface

The level of phosphate and protein in a healthy control mouse at 10d varied across the trabecular bone surface, measuring −7.7±2.9% and −6.9±2.7% lower at the peripheral edges, as compared to the total phosphate and protein through the bone closer to the center (p<0.05). Further, mineralization at these edges was −3.2±1.0% lower than the average level throughout the bone strut (p<0.05). Even following irradiation at 10d, the discrepancy in the phosphate, protein, and level of mineralization between the trabecular surface and the body of each bone strut remained similar, with a −6.6±5.0%, −6.5±3.0%, and −3.7±2.1% lower phosphate, protein, and mineralization on the periphery as compared to the total bone, respectively (p<0.05).

### Alteration in Mechanical Properties of Trabecular Bone Following Radiation

**Figure 3:**
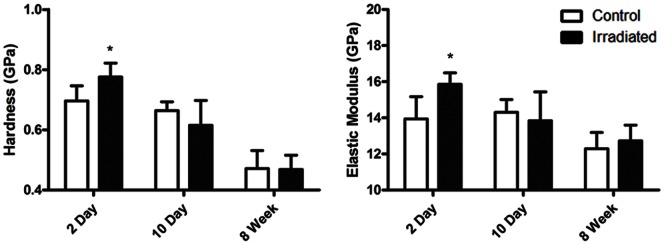
Mechanical properties of trabecular bone at 2d, 10d, and 8w following irradiation. As early as 2d following irradiation, alterations in the trabecular bone led to an increase in hardness and elastic modulus which was no longer present at 10d. *p<0.05 compared to control.

As early as 2d following radiation exposure, the elastic modulus of trabecular struts from irradiated mice were 13.7±4.5% higher than control (Irradiated: 15.9±0.6 GPa; Control: 13.9±1.2 GPa), while hardness was 11.5±6.6% higher (Irradiated: 0.78±0.05 GPa; Control: 0.70±0.05 GPa) as compared to control (p<0.05). Even though there was significant trabecular bone loss at 10d, the mechanical properties of the trabeculae from irradiated mice were no longer different from the control at 8w (Fig. 3). Whole bone testing though showed no differences in ultimate force and stiffness between irradiated and control mice at 10d.

### Collagen Type-I Release into Circulation

As early as 2d following irradiation, there is a 123% increase in CTX-I in the plasma of irradiated mice compared to control, with 4.1 ng/ml and 9.2 ng/ml of CTX-I in the control and irradiated mice, respectively (Average OD: Irradiated = 0.48±0.28, Control = 0.92±0.05; p<0.05). By 10d, the level of CTX-I in the plasma for irradiated mice remained 100% elevated, at 5.0 ng/ml and 10 ng/ml of CTX-I in the control and irradiated mice, respectively (Irradiated = 0.45±0.25 OD; Control = 0.78±0.19 OD; p<0.05).

### Increased Osteoclast Activity Following Irradiation Contributes to Bone Loss Even After Marked Depletion and Proliferative Capability of the Bone Marrow Progenitor Pool

**Figure 4:**
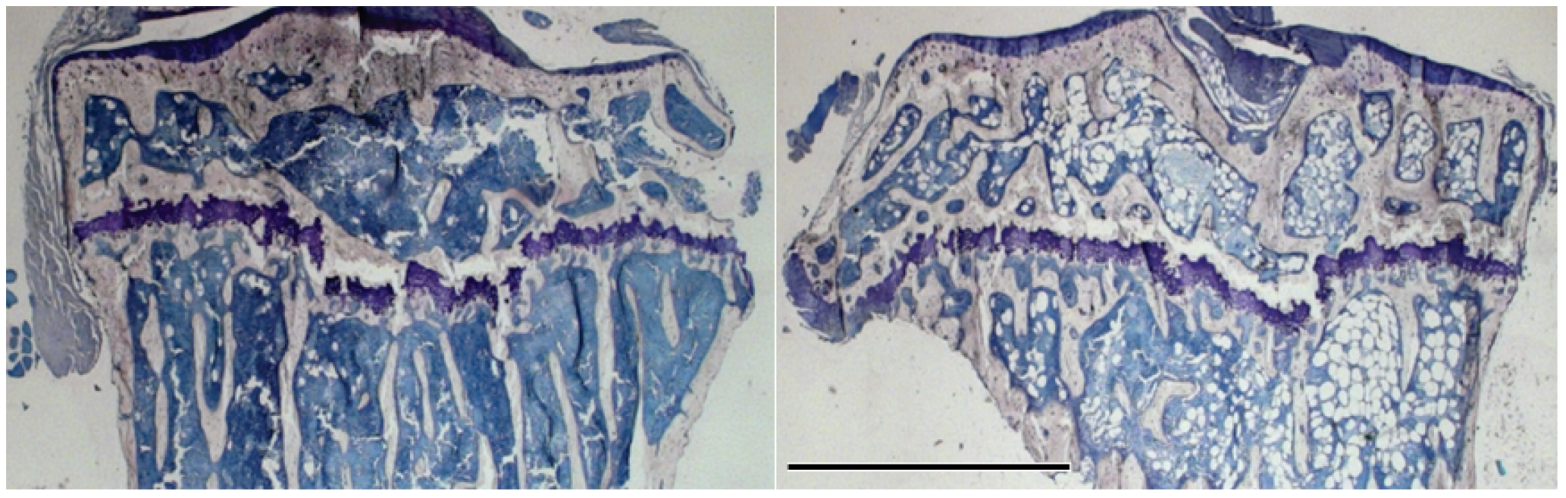
Light microscope image of mouse tibiae stained with Modified Wright Giemsa at 10d following irradiation. Image on the left is a control, and on the right is an irradiated mouse. The empty spaces in the marrow of the irradiated bone correspond to the rapid infiltration of fat cells, a consequence of which is that there is less space for immune cells to occupy the bone marrow space. Scale bar represents 1 mm.

**Figure 5:**
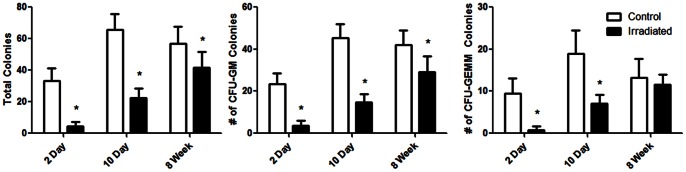
Total number of colony forming cells. CFU-GM, and CFU-GEMM in irradiated mice and age-matched control. Although the cell populations in irradiated mice remain depleted at 10d, they are more proliferative than irradiated cells at 2d and showed greater improvement by 8w. *p<0.05 compared to age-matched control.

At 2d following irradiation, the total number of cells in the bone marrow of irradiated mice declined by 65±11%, and remained 64±9% depleted at 10d [17]. This cellular depletion at 10d was accompanied by adipocyte infiltration into the marrow, with 6.2±2.9% of the bone marrow compartment occupied by adipocytes compared to only 0.58±0.40% in the control (p<0.05; Fig. 4). By 8w, the adipocytes in the bone marrow remained elevated and had 4.2±0.9% of their bone marrow composed of adipocytes compared to only 1.7±1.1% in the control (p<0.05). The total number of colony forming cells in irradiated mice was also markedly suppressed, showing an −86±8% and −66±9% decline at 2 & 10d relative to control (Fig. 5), indicating a marked reduction in the ability of cells to proliferate. At 2d, the number of CFU-GEMM and CFU-GM were −92±8% and −84±10% lower than control, whereas at 10d, there were −62±11% and −68±9% fewer CFU-GEMM and CFU-GM than control, demonstrating that at 10d the cells remaining were depressed relative to control, but more capable of proliferating than those measured at 2d following irradiation. By 8w, there were still −30±18% fewer CFU-GM cells than control, but the CFU-GEMM were no longer different (p = 0.32). Unlike the decline seen in these hematopoietic progenitor cells, at 2d TRAP5b concentrations in the plasma, used as a measure to quantify osteoclast activity and bone resorption [31], rose 43±35% in the irradiated mice compared to the control (p<0.05) [17]. The TRAP5b levels remained elevated by 24±33% at 10d (p = 0.06).

## Discussion

The rapid and extensive damage to bone as caused by irradiation, markedly diminishing bone quantity and quality and therefore elevating risk of fracture, appears to be the product of two distinct pathways: that driven by biologic processes orchestrated by elevated osteoclastic activity, and that facilitated by physicochemical processes enabled by compositional and material alterations in the composition of the matrix. The physicochemical processes described here refer to the physical and chemical changes to the bone matrix caused directly by the γ-rays. While two days post-irradiation revealed no measurable change in bone quantity as measured by microCT, a −4.3±4.8% (p = 0.07) trend towards a decline in the degree of mineralization of the trabecular bone, defined as the ratio of the phosphate/protein components, was already apparent. Analyzing the protein and phosphate peaks by FTIRI analysis showed this decline in mineralization to be due primarily to a decrease in the inorganic matrix component, representing hydroxyapatite, emphasizing a critical alteration to the material composition immediately following radiation exposure.

Despite a decrease in mineralization, change in the bone’s matrix properties as defined by nanoindentation revealed a 13.7±4.5% *increase* in elastic modulus, and 11.5±6.7% *elevation* in hardness in irradiated versus control mice at 2d. These changes occurred prior to visual bone loss. However, considering the rapid decline in bone quantity and mineralization seen at 10d, the elevation in material properties observed as early as 2d suggests that alterations in the matrix composition – specifically the relative increase of collagen relative to hydroxyapatite - makes it more susceptible to erosion, either through resorption achieved by biologically driven (cellular) processes, or by physical attrition, achieved independent of cellular actions. While certainly an extrapolation, perhaps the increase in collagen relative to mineral, as is evident in diseases such as osteomalacia [32], imply why that bone, too, is more susceptible to rapid loss of matrix.

Collagen has been shown to be more vulnerable to irradiation as compared to the inorganic phase of the matrix [14], [33], with gamma radiation compromising the collagen structure first by radiolysis of water molecules, which then leads to free radicals and increased cross-linking within the collagen fibrils [14]. The destruction of the organic phase – while contributing to a rise in measured modulus and hardness - somehow makes the matrix more susceptible to erosion *despite* the parallel devastation to the osteoclast precursor pool. Perhaps this is enabled by ionizing radiation mediated protein side chain decarboxylation from the phosphate groups bound to the hydroxyapatite crystals [34]. While we were unable to determine the actual physicochemical process of dissolution that were measured here, it does suggest a need to consider chemical – in concert with biologic – processes as contributing to the bone loss, and thus chemical – as well as biologic – strategies to slow this erosion.

By 10d, not only was there a significant depletion of trabecular bone in the irradiated mice, the level of tissue mineralization had declined by −5.8±3.2% relative to control. A question remained, however, whether the decline in tissue mineralization was uniform across the trabecular bone, or if only the periphery of the trabeculae was that which bore the brunt of the irradiation injury. Using the capacity of FTIRI to spatially map mineralization throughout a trabecular strut [35], it became evident that the decline was seen throughout the matrix at both 2d and 10d, implicating the ability of ionizing radiation to instigate physical damage through the trabecular bone struts, and providing further evidence that the increase in osteoclast activity at the bone surface was not the sole contributor to a decline in apparent bone density. However, the PMMA mask created was a limitation to this analysis because it excludes a ∼10 µm region of the trabecular bone surface, which is the region of greatest PMMA infiltration.

It is also important to consider that the bone marrow hematopoietic populations were largely extinguished at 2d, showing little recovery at 10d, reinforcing a conclusion that the loss of bone quantity was not achieved strictly by a biologic process alone. The CFU assay also quantifies the multipotential hematopoietic progenitor cells, and even after bone marrow cells were plated at equal concentrations, there were −86±8% and −66±9% fewer total CFUs at 2d and 10d compared to the control, exemplifying that hematopoietic cells of irradiated mice had inferior proliferative capabilities. The damage to the bone marrow was further exacerbated by immense adipocyte infiltration into the marrow seen at 10d, leading to the inhibition of hematopoietic stem cells necessary to maintain the bone marrow environment [36]. These adipocytes, derived from mesenchymal stem cells, were most likely capable of populating the bone marrow because of the massive deficit of other hematopoietic cell phenotypes [17]. TRAP5b expression though was markedly elevated at 2d, indicating increased osteoclast activity in the irradiated mice as compared to control even as the hematopoietic cells were depleted. Nevertheless, 2d is likely an insufficient period of time for osteoclasts to initiate bone destruction, with at least a 7d period required to increase osteoclast activity and numbers and to induce visible bone resorption in the mouse [37].

FTIRI analysis shows that the inorganic matrix in trabecular bone is more susceptible to radiation damage than the organic matrix following sub-lethal exposure, yet the cortical bone remains intact. The morphology of the two structures is very different, with more surface to volume in trabeculae, while the chemical composition of trabecular and cortical bone are also distinct, as cortical bone is much more mineralized, primarily due to the greater amount of the inorganic matrix. Therefore, it is likely that trabecular bone is more susceptible to radiation induced damage because of its greater surface area and less mineralized properties, leading to a surface more susceptible to dissolution. On the other hand, since the cortical bone is not as chemically sensitive to irradiation compared to trabecular bone, three point bending did not show differences in stiffness and ultimate force between irradiated and control mice at 10d, as these mechanical tests are primarily calculating the mechanical strength of the cortical bone.

While bone quantity had not recovered by 8w, likely due to the inability of the bone marrow to repopulate cellular populations (Fig. 5), irradiated mice exhibited some repair of the quality of the matrix as seen by chemical composition. Indeed, FTIRI analysis showed a 5.8±5.1% *increase* in the level of tissue mineralization of irradiated mice as compared to control (p<0.05), indicating that those trabeculae that remained had restored tissue mineralization properties to the point that they actually exceeded a healthy age-matched control mouse. However the structural properties within the remaining trabeculae were not identical between control and irradiated mice, as seen with lower crystallinity in irradiated mice. The smaller crystals in irradiated mice may be attributed to their more rapid bone turnover rates [17], leaving an insufficient period for their crystals to fully mature. By 8w, the hardness and elastic moduli were reduced for even the control mice, likely due to an age-related decline in mechanical properties [38], [39]. No differences in mechanical properties, however, were evident between the irradiated mice and age-matched control by 8w. This reparative process is perhaps best explained by the partial reconstitution of the bone marrow cell populations, as well as the increases in mineral apposition rate and bone formation rate of irradiated mice compared to control at 5w following radiation exposure [17]. This implies that osteoblasts are active in order to compensate for bone deterioration, but are incapable of restoring the structural damage.

Numerous studies have shown the deleterious impact of irradiation on bone [11], [40], but this critical outcome has most typically been presumed a result of elevated osteoclast activity. Here, however, we show that a 5 Gy dose of γ-irradiation, a relatively low dose when compared to those used for implant sterilization, can quickly alter both the chemical and physical makeup of the bone. These changes are likely the result of both biological and physicochemical contributions from osteoclasts and the radiation source. It is important to point out that those patients undergoing tumor ablation – who may receive localized doses greater than 60 Gy [16], [41], and bone marrow transplant patients subject to radiation doses greater than 10 Gy [15], are at far greater lifetime risk of fractures due to their bone deterioration. Perhaps the use of pharmacologic agents, such as bisphosphonates, which increase the total mineralization across the bone tissue [42], prior to radiation exposure could help protect bone quantity and quality, but non-intuitively through a process related to protecting the mineral rather than defeating the osteoclast.

### Conclusion

A rapid decline in tissue mineralization was seen throughout trabecular morphology of irradiated mice, which preceded a marked collapse of bone quantity. A parallel *increase* in matrix modulus and hardness, when considered in light of the devastation of the cellular population, suggests that bone loss is somehow facilitated by physicochemical changes in the composition of the matrix independent of a hampered biologic state. Ultimately, this study suggests that the bone loss following sub-lethal doses of irradiation, whether it is from intentional exposure used for medical treatments or accidental exposure from environmental disasters, is a result not only from biological processes, but by, chemical and physical changes to the composition of the matrix.
